# Pedicle subtraction osteotomy for the corrective surgery of ankylosing spondylitis with thoracolumbar kyphosis: experience with 38 patients

**DOI:** 10.1186/s12891-022-05693-z

**Published:** 2022-07-30

**Authors:** Haopeng Luan, Kai Liu, Alafate Kahaer, Yao Wang, Weibin Sheng, Maierdan Maimaiti, Hailong Guo, Qiang Deng

**Affiliations:** 1 Department of Spine Surgery, The First Affiliated Hospital of Xinjiang Medical University, Urumqi, 830054 Xinjiang China; 2 Department of Trauma and Microreconstructive Surgery, The First Affiliated Hospital of Xinjiang Medical University, Urumqi, 830054 Xinjiang China

**Keywords:** Ankylosing spondylitis, Pedicle subtraction osteotomy, Spine sagittal morphology, Sagittal parameter, Thoracolumbar kyphosis

## Abstract

**Objective:**

To evaluate the sagittal parameters and clinical outcome of pedicle subtraction osteotomy (PSO) for the treatment of ankylosing spondylitis (AS) combined with thoracolumbar kyphosis.

**Methods:**

The clinical data of 38 patients with AS combined with thoracolumbar kyphosis who underwent PSO were enrolled and divided into the lumbar lordosis group and the lumbar kyphosis group according to the preoperative sagittal morphology. They were subdivided into the lumbar lordosis T12 group, lordosis L1 group, kyphosis L2 group, and kyphosis L3 group. The spine sagittal parameters were compared between the preoperative and the postoperative. Outcome evaluation was performed by the Japanese Orthopedic Association (JOA) score, visual analogue scale (VAS), and the Oswestry Disability Index (ODI).

**Results:**

A total of 38 patients with AS combined with thoracolumbar kyphosis were successfully treated by PSO, with a mean follow-up time of 26.9 ± 11.9 months. There were 30 males and 8 females with a mean age of 41.6 ± 7.1 years. Twenty patients consisted in the lumbar lordosis group and 18 patients in the lumbar kyphosis group. GK, SVA, and CBVA were improved significantly (*P* < 0.05) at the final follow-up between the lumbar lordosis T12 group and the L1 group. Patients in the lumbar kyphosis L2 group and L3 group all received satisfactory, including LL, GK, and SVA (*P* < 0.05). There was no statistically significant difference in the preoperative TK, GK, SVA, PT, and PI between the lumbar lordosis and lumbar kyphosis groups (*P* > 0.05). Postoperative complications occurred in three cases.

**Conclusion:**

PSO was a practical method for the treatment of patients with AS combined with thoracolumbar kyphosis. PSO at L3 was recommended to be selected for the lumbar kyphosis to obtain greater SVA correction. CBVA of single-segment PSO may be significantly lower than the two-segment PSO in the management of patients with kyphosis of lower CBVA.

## Introduction

Ossification of spinal joints and ligaments, kyphotic deformity, and global spinal imbalance are the main trademarks of ankylosing spondylitis (AS), which is a chronic inflammation involving the mid axis of the spine [1]. In the advanced stage, patients with AS (approximately 30% of them with various degree of kyphosis) may experience back pain, insomnia, the anterior tilt of the trunk, abnormal gait, and even complicated digestive complications, which significantly affects the quality of life [2–4]. Correction of deformity, reducing the incidence of complications caused by abnormal posture, and improving quality of life are the main goals of surgery in the treatment of AS combined with thoracolumbar kyphosis [5]. Up to date, pedicle subtraction osteotomy (PSO) has been recommended widely by previous studies to restore the spinal sagittal sequence, since its simply anatomic structure of surgical approach, and effective deformity correction [6–8].

Although the satisfactory clinical outcomes of pedicle subtraction osteotomy (PSO) in the management of AS with thoracolumbar kyphosis, the level of osteotomy is a key point in the surgical procedure [9, 10]. Roussouly et al. [11] reported a series of 160 volunteers' spines evaluated by the special computer application and concluded that the lumbar lordosis was mainly dependent on the L3 to L5 vertebrae. However, Qian et al. [3] presented a consecutive series of 106 patients with ankylosing spondylitis combined with thoracolumbar kyphosis and pointed out that the L1 osteotomy conducted at the apex of the lordosis may receive satisfactory correction results. Further, Van Royen et al. [12] reported a total of 22 patients with progressive spinal kyphosis due to ankylosing spondylitis successfully treated by a closing-wedge posterior vertebral osteotomy with partial corpectomy of L4 and transpedicular fixation, concluded that the osteotomy level should be selected at the lower lumbar for the more effective sagittal vertical axis (SVA) correction. Hence, there is no definitive conclusion on the level of PSO for AS with lumbar lordotic or kyphotic deformity. In this study, the purpose was to evaluate the sagittal parameters and clinical outcome of pedicle subtraction osteotomy (PSO) for the treatment of ankylosing spondylitis (AS) combined with thoracolumbar kyphosis.

## Methods

The clinical data of patients with AS combined with thoracolumbar kyphosis managed by PSO between January 2008 and January 2020 at our hospital were retrospectively analyzed. The study was approved by the Ethics Committee of our hospital, and written informed consent from participants was received. The inclusion criteria were as follows: patients diagnosed with AS according to the modified New York criteria; thoracolumbar kyphosis certified by imaging films; treated by PSO surgery; followed-up time ≧ 12 months. The exclusion criteria included: incomplete medical records, poor compliance, or other treatments performed.

Global spine radiography was conducted on all patients to observe the sagittal morphology. According to the lumbar lordosis (LL), the patients were divided into two groups the lumbar lordosis group (LL < 0°) and the lumbar kyphosis group (LL > 0°). In particular, the deformity of vertebral bodies were identified based on sagittal parameters measured by radiography for the preparation of one-level PSO surgery [1]. The patients were subdivided into the lumbar lordosis T12 group, L1 group, L2 group, and L3 group.

### Surgical procedure

With the achievement of the effects of general anesthesia, the patient was placed in a prone position with pillows on both sides of the trunk so that the abdomen and chest can avoid pressure. The surgical area was routinely disinfected and covered with towels. The posterior median incision was made, and the skin and subcutaneous tissue were cut in layers. The latissimus dorsi and the thoracic dorsal fascia were cut, and the paravertebral muscles were peeled off under the spinous periosteum to reveal the area of kyphosis and the site requiring internal fixation. The muscle and tissue behind the vertebral body were carefully stripped to the lateral edge of the zygapophyseal joint, and the position of the preoperative planned osteotomized vertebra (OV) was fluoroscopically located with a C-arm X-ray machine, and a pedicle puncture was conducted. With the confirmation of the well-positioned nail path, pedicle screws were inserted in at least three segments of the cephalad and caudal sides of the OV, respectively. PSO was conducted to remove the lamina of the apical vertebra, and fornix decompression was conducted in the upper and lower laminae. Under rinsing with normal saline, a grinding drill was adopted to remove part of the OV through bilateral pedicles. While keeping the inner wall of the pedicle intact as much as possible, the drill was adopted to thin the outer and anterior walls of the vertebral body laterally and anteriorly and penetrate through the middle of the vertebral body internally. Finally, the bone of the posterior wall of the vertebral body was removed with the adoption of tools such as nucleus pulposus bite forceps, laminae bite forceps, and scrapers to create a narrow wedge-shaped space inside the vertebral body. After completing the osteotomy, the sponge pad on the abdomen was removed, and gradual closure of the osteotomized space was visible. A titanium rod with appropriate length was pre-bent, and the osteotomized end was closed gradually with appropriate pressure between screws. A wake-up test was performed at closure, and the patient had good movement of both lower extremities. The posterior interlaminar cortex was removed, and interlaminar fusion was conducted after grinding the spinous process and the bone of the vertebral plate into a granular form. Fluoroscopy with a C-arm X-ray machine was conducted to observe the closure of the osteotomized surface and osteotomized angle, and the dural sac of the spinal canal and the corresponding nerve roots were re-examined to confirm that there was no compression and injury. The surgical field was flushed repeatedly with normal saline, and a drainage tube was inserted. The incision was closed layer by layer.

### Postoperative management and outcome evaluation

The drainage tube was removed when the daily drain volume was < 50 ml. Usually, patients were encouraged to active mobility with the help of a brace after 4 to 5 postoperative days. Sagittal parameters evaluation was conducted by radiography at 1, 3, 6, and 12 postoperative months including global kyphosis (GK) angle, thoracic kyphosis (TK) angle, lumbar lordosis (LL) angle, the sagittal vertical axis (SVA), sacral slope (SS), pelvic tilt (PT), pelvic incidence (PI), and chin-brow vertical angle (CBVA). Outcome evaluation was performed by the Japanese Orthopedic Association (JOA) score, visual analogue scale (VAS), and the Oswestry Disability Index (ODI).

### Statistical analysis

The SPSS 21.0 software (Chicago, IL, USA) was applied for statistical analysis. Continuous variables were expressed as mean ± standard deviation and analyzed by the Kirmogrov-Smirnov test for normality assessment. Differences (LL < 0° group vs LL > 0°group) were analyzed by unpaired t-test or Mann–Whitney U test. Categorical variables were analyzed by chi-square test. *P* < 0.05 was considered a statistical significance.

## Results

**Fig. 1:**
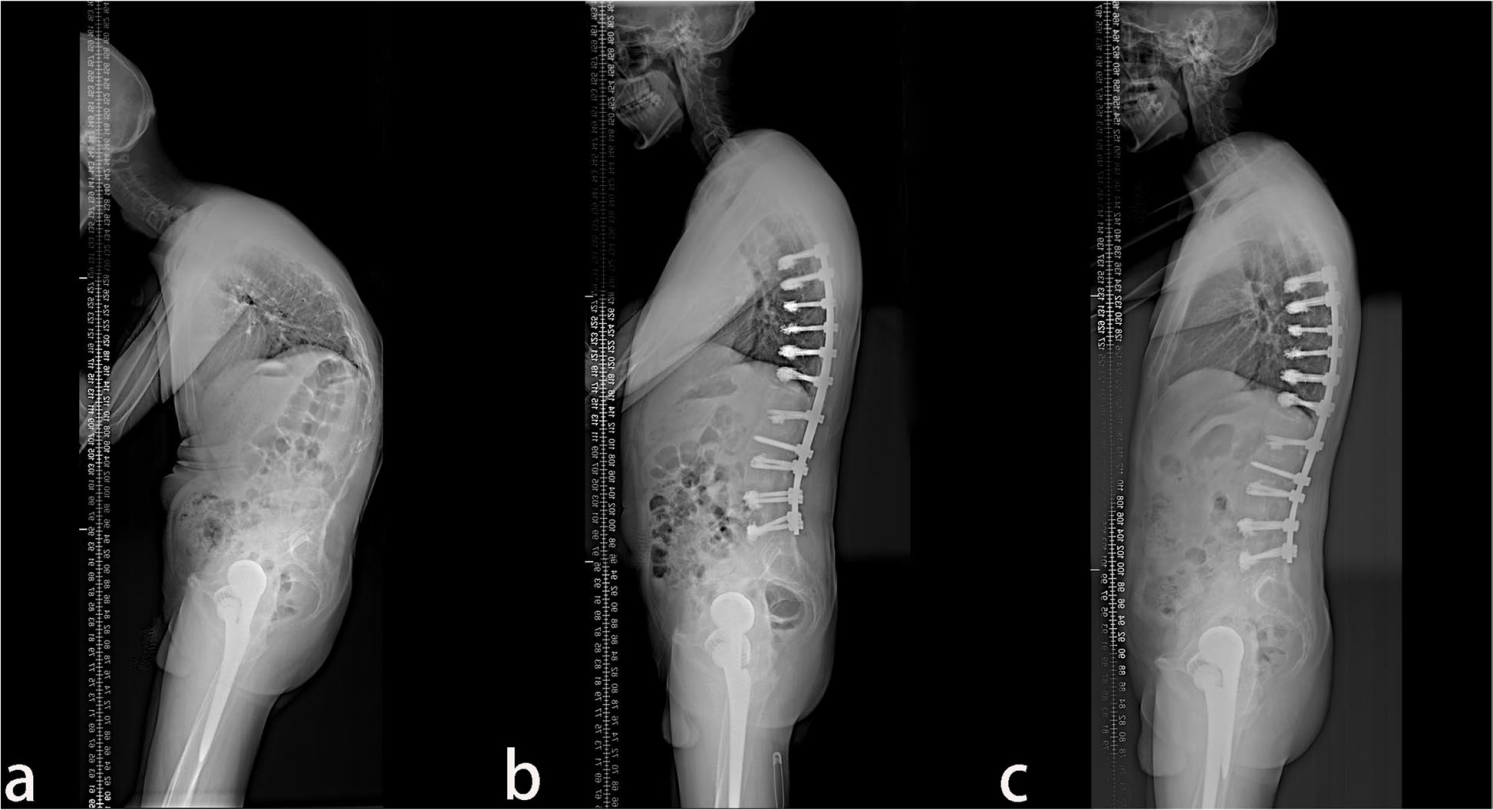
A 31-year old male with AS. **a** The preoperative X-ray showed that the parietal vertebrae were located at L1, which belonged to the lumbar lordosis group. **b**) Single-segment pedicle subtraction osteotomy (PSO) was conducted at T12. Lumbar lordosis, global kyphosis, and sagittal vertical axis improved from the preoperative –44°, 86°, and 162.29 mm to –45°, 47°, and 24.41 mm, respectively. **c**) At the last follow-up in the 16th-month post-operation, no apparent orthopedic loss was observed, and lumbar lordosis, global kyphosis, and sagittal vertical axis were –41°, 53°, and 30.67 mm, respectively

**Fig. 2:**
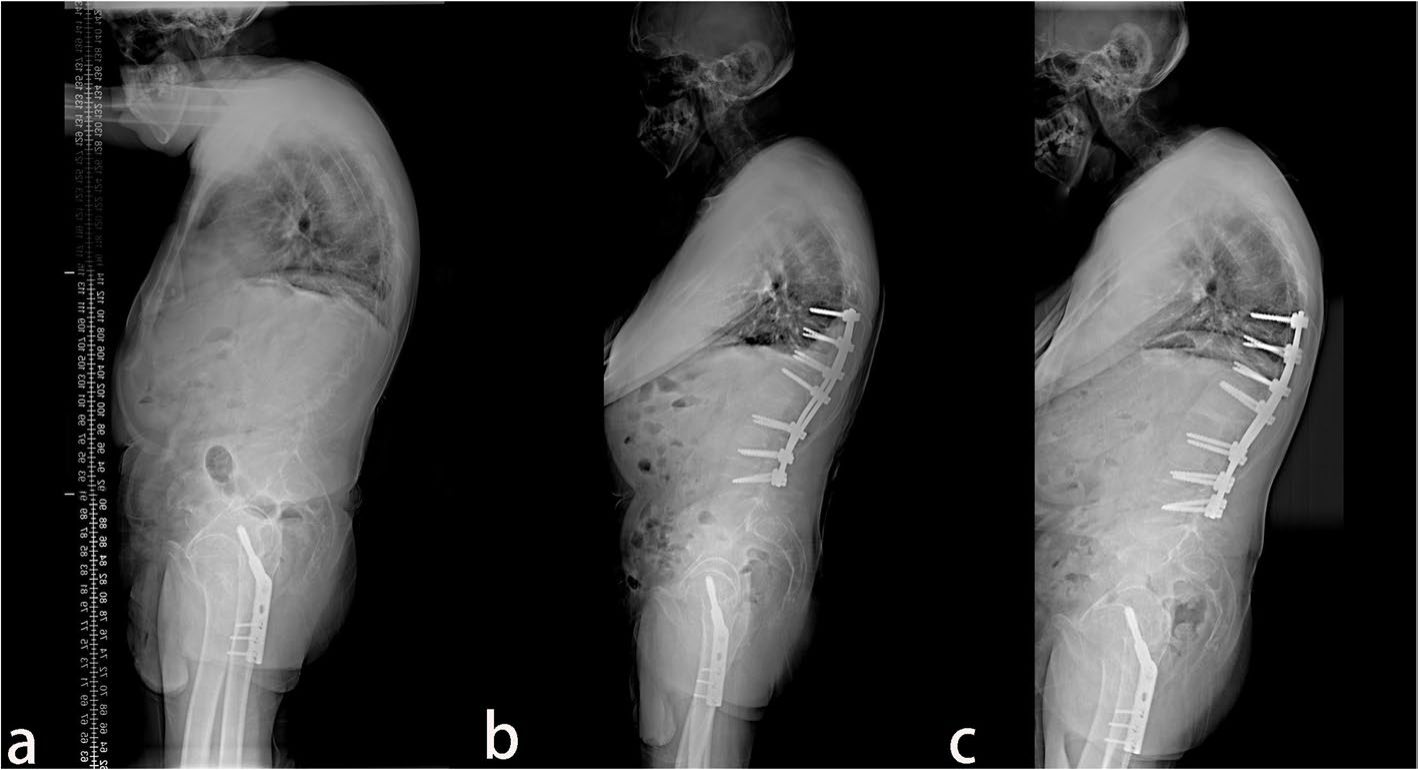
A 49-year old male patient with AS. **a** The preoperative X-ray showed that the parietal vertebrae were located at L2, which belonged to the lumbar lordosis group. **b** Single-segment pedicle subtraction osteotomy (PSO) was conducted at L1. Lumbar lordosis, global kyphosis, and sagittal vertical axis improved from the preoperative –25°, 79°, and 104.21 mm to –53°, 61°, and 30.34 mm, respectively. **c** At the last follow-up in the 30th-month post-operation, no apparent orthopedic loss was observed, and lumbar lordosis, global kyphosis, and sagittal vertical axis were –49°, 51°, and 31.76 mm, respectively

**Fig. 3:**
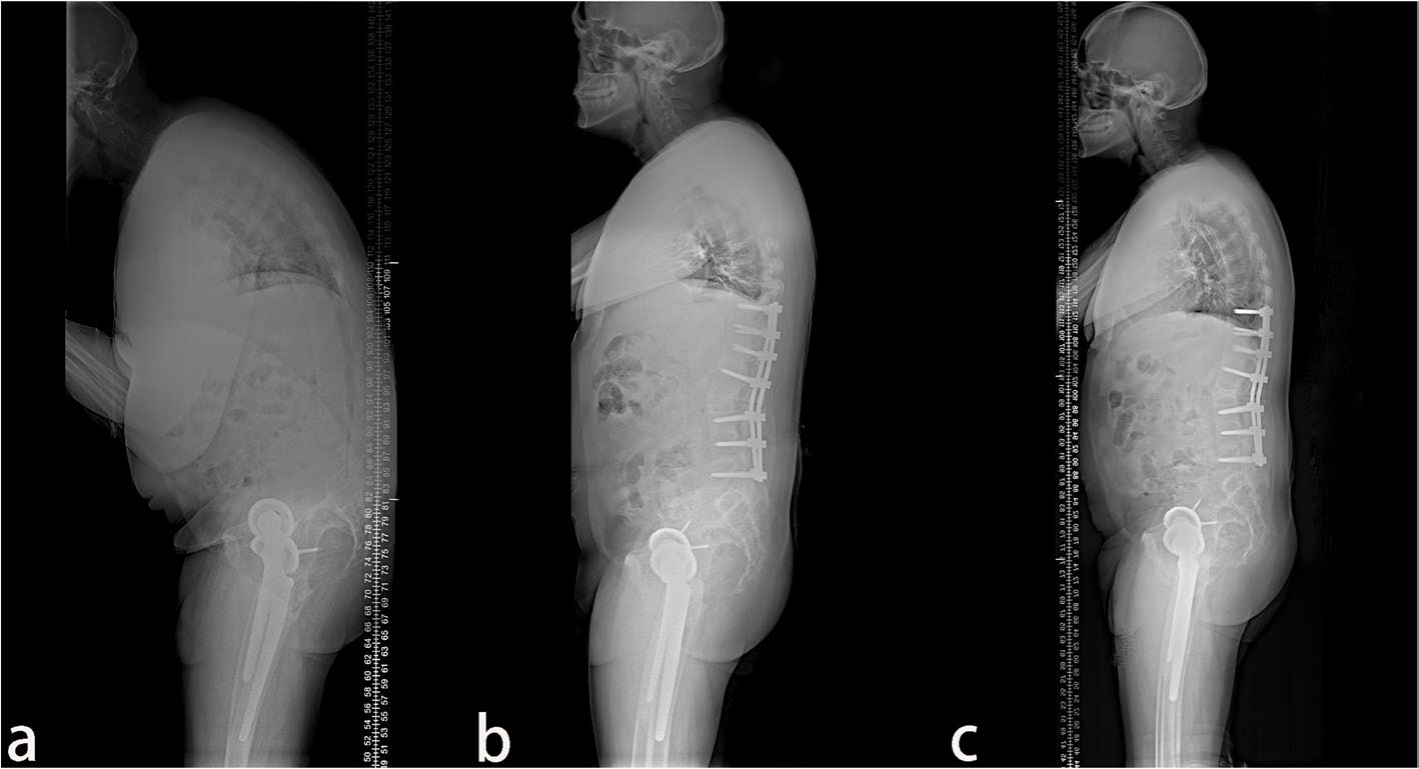
A 39-year old male patient. **a** The preoperative X-ray showed that the parietal vertebrae were located at T10, which belonged to the lumbar kyphosis group. **b** Single-segment pedicle subtraction osteotomy (PSO) was conducted at L2. Lumbar lordosis, global kyphosis, and sagittal vertical axis improved from the preoperative 8°, 78°, and 317.39 mm to –47°, 53°, and 60.47 mm, respectively. **c** At the last follow-up in the 28th-month post-operation, no apparent orthopedic loss was observed, and lumbar lordosis, global kyphosis, and sagittal vertical axis were –56°, 48°, and 55.56 mm, respectively

**Fig. 4:**
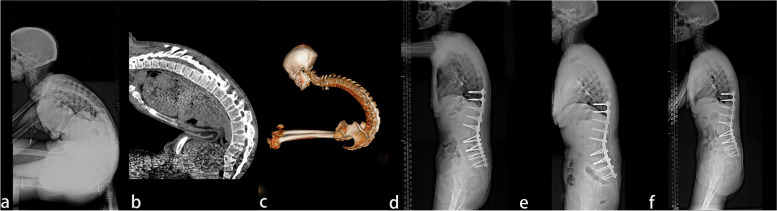
A 46-year old female patient. **a**, **b**, **c** The preoperative X-ray and 3-D CT reconstruction showed that the parietal vertebrae were located at T11/T12, which belonged to the lumbar kyphosis group. **d** Smith-Petersen osteotomy was conducted at L1–L2, L4–L5, and L5–S1, together with single-segment pedicle subtraction osteotomy (PSO) at L3. Lumbar lordosis, global kyphosis, and sagittal vertical axis improved from the preoperative 44°, 105°, and 257.39 mm to –44°, 53° and 55.47 mm, respectively. e) One-year post-operation, lumbar lordosis, global kyphosis, and sagittal vertical axis were –45°, 60°, and 47.53 mm, respectively. f) At the last follow-up at the 37th-month post-operation, no apparent orthopedic loss was observed, and lumbar lordosis, global kyphosis, and sagittal vertical axis were –47°, 61°, and 48.56 mm, respectively

A total of 38 patients with AS combined with thoracolumbar kyphosis were successfully treated by PSO, with a mean follow-up time of 26.9 ± 11.9 months. There were 30 males and 8 females with a mean age of 41.6 ± 7.1 years. Twenty patients consisted in the lumbar lordosis group and 18 patients in the lumbar kyphosis group, with a mean operation time of 289.4 ± 123.2 min and 267.8 ± 96.8 min (*P* > 0.05), respectively. The patients were further subdivided into the lumbar lordosis T12 group (Fig. 1) with 11 patients, the lumbar lordosis L1 group (Fig. 2) with 9 patients, the lumbar kyphosis L2 group (Fig. 3) with 10 patients, and the lumbar kyphosis L3 group (Fig. 4) with 8 patients.

The sagittal parameters and clinical symptoms were improved significantly at the final follow-up in all patients (Table 1). GK, SVA, and CBVA were improved significantly (*P* < 0.05, Table 2) at the final follow-up between the lumbar lordosis T12 group and the L1 group. Patients in the lumbar kyphosis L2 group and L3 group all received satisfactory, including LL, GK, and SVA (*P* < 0.05, Table 3). However, there was no statistically significant difference in the preoperative TK, GK, SVA, PT, and PI between the lumbar lordosis and lumbar kyphosis groups (*P* > 0.05).

**Table 1:** Comparison of preoperative and postoperative sagittal parameters and function recovery between the lordosis group and kyphotic group

Variables		Lordosis group	Kyphotic group	*P* value
LL(°)	Preoperative	-30.9 ± 19.4	13.1 ± 11.1	< 0.001
	Final follow-up	-45.5 ± 7.5	-43.2 ± 8.4	0.431
	*P* value	0.002	< 0.001	
TK(°)	Preoperative	50.7 ± 9.9	51.7 ± 7.5	0.954
	Final follow-up	49.3 ± 8.7	49.2 ± 10.3	0.535
	*P* value	0.684	0.536	
GK(°)	Preoperative	76.2 ± 18.4	87.5 ± 31.2	0.352
	Final follow-up	51.5 ± 6.8	54.6 ± 12.3	0.284
	*P* value	< 0.001	0.005	
SVA(mm)	Preoperative	169.1 ± 89.1	218.5 ± 67.8	0.142
	Final follow-up	53.2 ± 19.1	65.5 ± 17.8	0.075
	*P* value	< 0.001	< 0.001	
PT(°)	Preoperative	39.1 ± 13.4	37.5 ± 15.2	0.813
	Final follow-up	24.8 ± 9.6	23.4 ± 11.1	0.956
	*P* value	< 0.001	< 0.001	
PI(°)	Preoperative	65.6 ± 16.5	52.6 ± 17.1	0.254
	Final follow-up	59.8 ± 15.1	54.1 ± 12.1	0.501
	*P* value	0.063	0.665	
SS(°)	Preoperative	26.4 ± 7.9	15.2 ± 9.5	0.002
	Final follow-up	34.8 ± 7.5	30.6 ± 6.4	0.073
	*P* value	0.004	0.001	
CBVA(°)	Preoperative	28.2 ± 17.1	34.4 ± 30.2	0.536
	Final follow-up	13.2 ± 7.5	13.6 ± 3.3	0.765
	*P* value	< 0.001	0.047	
JOA	Preoperative	13.4 ± 3.1	11.6 ± 3.1	0.646
	Final follow-up	23.0 ± 2.2	23.3 ± 2.3	0.406
	*P* value	< 0.001	< 0.001	
VAS	Preoperative	7.8 ± 0.9	7.1 ± 1.2	0.168
	Final follow-up	1.8 ± 0.5	1.6 ± 0.4	0.463
	*P* value	< 0.001	< 0.001	
ODI	Preoperative	32.9 ± 10.1	25.8 ± 8.2	0.128
	Final follow-up	16.1 ± 6.4	15.1 ± 6.9	0.865
	*P* value	< 0.001	0.001

**Table 2:** Comparison of preoperative and postoperative sagittal parameters and function recovery between the lordosis T12 group and L1 group

Variables		T12 group	L1 group	*P* value
LL(°)	Preoperative	-34.6 ± 23.1	-26.1 ± 13.2	0.570
	Final follow-up	-45.7 ± 7.9	-45.2 ± 7.5	0.582
	*P* value	0.134	0.002	
TK(°)	Preoperative	50.5 ± 11.9	50.9 ± 8.7	0.959
	Final follow-up	51.7 ± 6.7	49.7 ± 6.6	0.622
	*P* value	0.803	0.752	
GK(°)	Preoperative	83.2 ± 16.8	67.14 ± 17.1	0.009
	Final follow-up	51.7 ± 6.1	51.2 ± 8.2	0.981
	*P* value	< 0.001	0.009	
SVA(mm)	Preoperative	169.1 ± 108.2	168.9 ± 65.1	0.283
	Final follow-up	55.7 ± 20.1	49.8 ± 18.5	0.727
	*P* value	0.007	0.002	
PT(°)	Preoperative	34.8 ± 14.2	44.4 ± 10.7	0.331
	Final follow-up	23.4 ± 12.4	26.6 ± 4.3	0.238
	*P* value	< 0.001	0.020	
PI(°)	Preoperative	63.5 ± 20.1	68.3 ± 11.2	0.474
	Final follow-up	59.7 ± 19.1	59.8 ± 9.3	0.690
	*P* value	0.309	0.262	
SS(°)	Preoperative	28.4 ± 8.5	23.8 ± 6.9	1.000
	Final follow-up	35.8 ± 8.2	33.3 ± 6.5	0.435
	*P* value	0.046	0.004	
CBVA(°)	Preoperative	26.2 ± 18.1	31.1 ± 16.5	0.214
	Final follow-up	13.4 ± 10.1	12.8 ± 1.95	0.343
	*P* value	0.009	0.003	
JOA	Preoperative	13.8 ± 3.3	12.7 ± 2.8	0.934
	Final follow-up	23.8 ± 1.9	21.8 ± 1.9	0.849
	*P* value	< 0.001	0.002	
VAS	Preoperative	7.7 ± 0.9	7.9 ± 0.9	0.296
	Final follow-up	1.7 ± 0.5	1.9 ± 0.4	0.424
	*P* value	< 0.001	< 0.001	
ODI	Preoperative	30.3 ± 9.9	36.1 ± 9.9	0.588
	Final follow-up	17.2 ± 8.1	14.7 ± 3.4	0.659
	*P* value	0.010	0.034

**Table 3:** Comparison of preoperative and postoperative sagittal parameters and function recovery between the kyphotic L2 group and L3 group

Variables		L2 group	L3 group	*P* value
LL(°)	Preoperative	10.5 ± 8.1	16.1 ± 14.3	0.330
	Final follow-up	-45.1 ± 9.1	-41.1 ± 7.9	0.908
	*P* value	< 0.001	< 0.001	
TK(°)	Preoperative	50.4 ± 8.6	50.3 ± 9.4	0.755
	Final follow-up	49.6 ± 7.6	51.1 ± 4.8	0.911
	*P* value	0.767	0.920	
GK(°)	Preoperative	69.3 ± 25.5	109.2 ± 23.1	0.113
	Final follow-up	56.5 ± 15.2	52.4 ± 8.6	0.831
	*P* value	0.076	0.02	
SVA(mm)	Preoperative	190.2 ± 68.5	252.4 ± 54.5	0.796
	Final follow-up	62.8 ± 25.4	68.7 ± 23.9	0.408
	*P* value	0.004	< 0.001	
PT(°)	Preoperative	35.3 ± 14.2	40.1 ± 17.6	0.359
	Final follow-up	20.3 ± 10.9	27.1 ± 11.1	0.804
	*P* value	0.012	0.006	
PI(°)	Preoperative	51.3 ± 20.9	54.1 ± 13.5	0.776
	Final follow-up	52.1 ± 13.4	56.2 ± 11.4	0.561
	*P* value	0.896	0.143	
SS(°)	Preoperative	16.2 ± 9.8	14.1 ± 10.1	0.017
	Final follow-up	31.8 ± 7.4	29.2 ± 5.4	0.114
	*P* value	0.041	0.001	
CBVA(°)	Preoperative	23.8 ± 12.5	47.1 ± 41.4	0.786
	Final follow-up	15.1 ± 1.8	12.1 ± 4.1	0.699
	*P* value	0.163	0.023	
JOA	Preoperative	11.8 ± 2.1	11.4 ± 4.2	0.067
	Final follow-up	23.6 ± 2.7	22.8 ± 1.8	0.006
	*P* value	< 0.001	< 0.001	
VAS	Preoperative	6.9 ± 1.1	7.3 ± 1.4	0.481
	Final follow-up	1.6 ± 0.5	1.7 ± 0.4	0.553
	*P* value	< 0.001	< 0.001	
ODI	Preoperative	26.7 ± 8.6	24.9 ± 8.6	0.267
	Final follow-up	16.2 ± 6.1	13.6 ± 8.3	0.530
	*P* value	0.034	0.002	

There was no case with the sagittal displacement of the spinal vertebra. Postoperative complications occurred in three cases. Intraoperative dural tear combined with postoperative parietal aeration cerebrospinal fluid leak took place in one patient and recovered by prolonging the duration of bed rest. Delayed incision healing occurred in one patient since diabetes mellitus and was improved by regular glucose-lowering medication and oral antibiotics. Re-fracture of the internal fixation occurred in one patient and was successfully managed by revision surgery.

## Discussion

The key point for the management of AS combined with thoracolumbar kyphosis is to restore the sagittal balance, which should keep in consideration to determine the PSO level. The parietal or lower lumbar vertebrae are usually selected as the PSO level to obtain local and overall sagittal balance [12, 13]. Via published studies [14], better correction may be received by the PSO at the parietal vertebrae, while PSO at lower levels of the vertebral body may improve the safety of the surgical operation [12, 15]. Debarge et al. [16] suggested that the correction of pelvic spinal parameters was closely correlated with the PSO level. Chen et al. [15] compared the corrective effect of PSO at a different level for the treatment of AS combined with thoracolumbar kyphosis and concluded that PSO at the lower lumbar vertebrae could maximize the improvement of GK. However, Lafage et al. [9] reported that there was a stronger correlation between pelvic spinal parameters and the osteotomy angle than the PSO level. In this study, the L3 level was recommended for conducting the PSO. It was demonstrated that PSO in the treatment of AS combined with thoracolumbar deformity may receive a satisfactory postoperative functional recovery, including significant improvement in JOA, VAS, and ODI at follow-up (*P* < 0.05). The preoperative and postoperative LL and SS in the kyphosis group were higher than in the lordosis group (*P* < 0.05). However, CBVA brought out by single-segment PSO may be significantly lower than the two-segment PSO in the management of patients with kyphosis of lower CBVA.

Kyphosis deformity usually occurs at the thoracolumbar vertebrae [3, 17]. Previously, a relationship between the anatomical parameter of pelvic incidence and the SS, which strongly determines LL, was observed by Legaye et al. [13]. Thomasen et al. [1] firstly proposed the PSO for the treatment of AS with severe kyphosis and introduced the detail of PSO. Subsequently, Kim et al. [18] and Gupta et al. [19] reported that the SVA and LL could be improved significantly by the PSO with less injury to nerves and blood vessels, which had become one of the most common procedures to treat AS with kyphosis. Xu et al. [20] reported a series of 60 patients with AS kyphosis successfully treated by PSO, and the total spine correction was 43.2 ± 15.1° (single-level) and 60.6 ± 19.1° (two-level). Diao et al. [21] presented a study of 71 patients with AS combined with thoracolumbar kyphosis effectively managed by PSO, and double-level PSO should be considered when the correction angle was greater than 60°. In our cohort, the average correction in LL, GK, and SVA was 33°, 41°, and 107 mm, respectively, in the lumbar lordosis and lumbar kyphosis groups.

The spinal deformity was usually the sum of global imbalance and compensatory changes. For the thoracolumbar spine, most patients with AS involved two or more vertebral bodies, but AS combined with kyphotic deformity mostly presented with a single vertebral sagittal deformity. The preoperative and postoperative LL in the kyphosis group were higher than in the lordosis group in this study, and PSO was conducted at T12/L1 and L2/3 in the lordosis group and kyphosis group, respectively. In our experience, a longer force arm during osteotomy closure could be received by PSO at the lower lumbar, which allowed for maximum correction of LL and SVA. And SS in this cohort was significantly improved after surgery, which indicated that the posterior pelvic tilt was corrected after restoring the overall sagittal balance. In the comparison of the correction rates of PSO at different vertebrae levels, there was an upward trend in the correction rate of PSO as the osteotomy segment decreased from T12 to L3. And the fixation at S1 was often required for PSO at L3. However, Yao et al. [10] considered that fixation at S1 might be responsible for the decreasing ODI postoperatively. In this cohort, there were 8 patients with L3 kyphosis successfully treated by PSO. And the ODI at the final follow-up in the patients treated by PSO at L3 was 13.6 ± 8.3, without sagittal imbalance. As far as we were considering, greater improvement of LL and SVA could be received when PSO was applied at L3 vertebrae. And it was necessary to apply fixation at S1 for obtaining the stability of sagittal balance. It was generally recommended that PSO should be at or near the top of the kyphosis to acquire a harmony postoperative alignment with less risk of neurological injury.

Via previous articles [1, 5, 8], double-segment PSO or PSO combined with Smith-Petersen osteotomy (SPO) was recommended to treat patients with severe kyphosis (> 60°) to obtain a stable sagittal balance. In the present study, a 46-year-old male (Fig. 4) with a typical "folded knife dorsum" preoperatively possessed a CBVA of –60°, and an SVA of 257.39 mm combined with lumbar lordosis (LL > 45°). Preoperatively, the required osteotomy angle was calculated to be 86.5°, and SPO combined with PSO was performed. Briefly, SPO was conducted at L5–S1, L4–L5, L1–L2, and PSO osteotomy at L3, respectively, combined with internal fixation of T9–S1 vertebrae. In our cohort, postoperative CBVA, LL, GK, and SVA were greatly improved. In one postoperative year, a satisfactory spinal sagittal balance with good function recovery was received.

In the selection of PSO, the parietal vertebrae level is also an important factor to keep in consideration. It was suggested by previous studies [1, 19, 21] that PSO should be conducted at the parietal vertebrae because greater SVA correction could be obtained [22]. Chen et al. [15] reported a series of 78 patients with AS scheduled by parietal vertebrae osteotomy and showed satisfactory results with a mean kyphosis correction per segment of 34.5°. In this study, patients with lumbar lordosis usually combined with a kyphosis deformity in the thoracolumbar segmental junction area, where L1 was recommended to be selected for PSO. L1 was relatively close to the parietal vertebrae, which could achieve better correction of thoracolumbar lordosis, and reduce the risk of postoperative proximal junctional kyphosis (PJK) since less segment fixation.

Seven patients in this study were used to be performed hip arthroplasty (total hip arthroplasty, THA) before PSO. To our knowledge, spinal osteotomy and THA were recommended to treat patients with AS combined with thoracolumbar kyphosis and severe hip deformity [19, 20]. However, it remains controversial how to schedule the sequence of two surgical interventions. Zheng et al. [23] considered that lumbar PSO first might correct the sagittal plane posterior tilt of the pelvis and reduce the risk of prosthesis dislocation after THA. In contrast, Bhan et al. [24], Mou et al. [25], and Joshi et al. [26] suggested that THA should be performed firstly since its improves of the hip mobility and intraoperative positioning of PSO.

There were some limitations in the study. First, the present study was a small-sample-size retrospective study. Second, there was a lack of a clear treatment algorithm for patients with AS combined with thoracolumbar kyphosis. Therefore, a large-sample prospective study was needed to refine the classification of lumbar sagittal morphology in patients with AS.

## Conclusion

PSO was a practical method for the treatment of patients with AS combined with thoracolumbar kyphosis. Better preoperative sagittal alignment with the apices was usually located in the upper lumbar region of patients with LL. PSO at L3 was recommended to be selected for the lumbar kyphosis to obtain greater SVA correction. CBVA of single-segment PSO may be significantly lower than the two-segment PSO in the management of patients with kyphosis of lower CBVA.

## Data Availability

The data sets generated and analyzed during the current study are not publicly available due to restrictions on ethical approvals involving patient data and anonymity but can be obtained from the corresponding author on reasonable request.

## Abbreviations

AS: Ankylosing spondylitis
CBVA: Chin-brow vertical angle
GK: Global kyphosis
LL: Lumbar lordosis
ODI: Oswestry disability index
OV: Osteotomied vertebra
PI: Pelvic incidence
PSO: Pedicle subtraction osteotomy
PT: Pelvic tilt
SS: Sacral slope
SVA: Sagittal vertical axis
THA: Total hip arthroplasty
TK: Thoracic kyphosis
